# Evaluation of one-stop diagnosis and management at the collaborative national standardized metabolic disease management center

**DOI:** 10.3389/fendo.2025.1490131

**Published:** 2025-05-06

**Authors:** Huihui Yin, Shanshan Yu, Han Li, Chunhong Shi, Weiping Wang, Lili Men, Lihong Jia

**Affiliations:** ^1^ Department of Nursing, First Affiliated Hospital, Dalian Medical University, Dalian, China; ^2^ First Affiliated Hospital, Dalian Medical University, Dalian, China; ^3^ Department of Endocrinology and Metabolism, the First Affiliated Hospital of Dalian Medical University, Dalian, China

**Keywords:** collaborative healthcare, national standardized metabolic disease management center, diabetes, diagnosis and treatment workflow, management mode

## Abstract

**Objective:**

To evaluate the effectiveness of diagnosis and treatment at the collaborative National Standardized Metabolic Disease Management Center.

**Methods:**

A nationally standardized selection process was used to recruit the healthcare team, followed by standardized training and assessment. The management center was equipped with coordinated healthcare resources, and clear workflows and responsibilities were established. A quality control management model was implemented throughout all stages. A convenience sample of 452 patients treated at the center between January 2018 and July 2023 was selected. After one year of management, a self-comparison was conducted to evaluate the impact of one-stop diagnosis and management on patients’ weight, fasting blood glucose, glycated hemoglobin, blood pressure, blood lipids, diet, and exercise.

**Results:**

After one year of management, patients showed significant reductions in weight, BMI, waist circumference, and visceral fat compared to baseline data (*P*=0.000). Fasting blood glucose and glycated hemoglobin levels decreased significantly (*P*<0.0001), as did systolic and diastolic blood pressure (*P*<0.05). Triglycerides, low-density lipoprotein cholesterol, and total cholesterol levels also decreased significantly (*P*<0.05), but high-density lipoprotein levels did not change significantly (*P*=0.5298). Improvements were observed in dietary and exercise behaviors (*P*<0.05), though no significant change was observed in salt intake (*P*=0.648).

**Conclusion:**

The collaborative model at the National Standardized Metabolic Disease Management Center enhances comprehensive patient management. Combined with lifestyle guidance on diet and exercise, the model improves the prevention and control of glycemic and lipid metabolism indicators in diabetes patients.

## Introduction

The prevalence of diabetes has been steadily increasing due to societal progress and various influencing factors. According to the 2021 International Diabetes Federation (IDF) survey, 537 million adults worldwide have diabetes, with a prevalence rate of 10.5%. By 2045, this number is projected to rise to 738 million. In China, the number of individuals aged 20 to 79 with diabetes has grown from 90 million to 140 million over the past decade, marking a 56% increase. These statistics highlight the severity of the diabetes situation, underscoring the need for effective prevention and management as a major public health concern (1). Research indicates that type 1 diabetes accounts for 5.8% of the diabetic population, while non-type 1 diabetes makes up 94.2%, with type 2 diabetes constituting over 90% of all cases (2, 3). However, awareness, treatment, and control rates remain relatively low, emphasizing the need for improved diabetes management. Internationally, well-established chronic disease care models help patients control blood glucose levels by addressing lifestyle factors, modifying dietary habits, and adjusting exercise routines. These models also focus on preventing and managing complications, thereby reducing the risk of diabetes-related cardiovascular diseases (4, 5). With advancements in medical technology, these models have incorporated “Internet+” solutions, medical alliances, and multidisciplinary collaboration. This integration improves patient compliance, enhances disease control, and increases patient satisfaction (6, 7). Despite extensive research on diabetes management in China, no mature model with Chinese characteristics has been developed (8). In 2016, the National In 2016, the National Metabolic Disease Clinical Research Center, the Shanghai Institute of Endocrine and Metabolic Diseases, and Academician Ning Guang of the Chinese Academy of Engineering proposed and established the National Metabolic Management Center (MMC) (9). The MMC introduces a new model for diagnosing and managing metabolic diseases based on the core principles of “one center, one-stop service, one standard.” This model addresses the need for screening, diagnosis, management, and clinical research of metabolic diseases and their complex complications. It integrates scenarios, data, technology, and applications into a closed-loop management system, enabling comprehensive follow-up management of diabetes patients both inside and outside the hospital, online and offline. This approach improves the quality and efficiency of medical care, making patient visits more efficient and convenient. Based on the first MMC established at our hospital, this study explores the current standardized management model of the MMC. It focuses on the collaboration among doctors, nurses, and other healthcare professionals in managing glycemic and lipid metabolism indicators and the lifestyles of diabetic patients to evaluate the benefits of one-stop diagnosis and management for patients.

## Materials and methods

### Study subjects

The study included 452 diabetes patients who received MMC management at the First Affiliated Hospital of Dalian Medical University from January 2018 to July 2023 and completed a one-year follow-up according to standardized procedures. Patients were selected using a convenience sampling method. Inclusion criteria were as follows: (1) meeting the 1999 World Health Organization (WHO) diagnostic criteria for diabetes (10); (2) aged 18 to 75 years; (3) patients and their families could accept follow-up via the MMC APP, phone calls, text messages, or WeChat; (4) no cognitive or behavioral impairments, and able to communicate normally; (5) willing to participate in this study. Exclusion criteria were: (1) severe diabetes complications such as diabetic ketoacidosis, cardiovascular and cerebrovascular diseases, renal failure, or diabetic foot; (2) participation in other research projects; (3) loss to follow-up.

### Methods

#### MMC healthcare personnel and equipment setup

The MMC management team in this study comprised 5 doctors and 2 nurses, with the head nurse and department director overseeing quality control and outcome evaluation. All team members underwent training on the Standard Operating Procedures (SOP) established by MMC. The hospital offers specialized outpatient clinics for diabetes and metabolic diseases, a nursing clinic, and a diabetes examination room, where staff are responsible for diagnosis, data registration, appointment scheduling, follow-up management, and health education.

The hardware and software setup is based on an internet-enabled platform that integrates hospital and external data. Relevant diagnostic equipment, computers, and synchronized mobile apps for both healthcare providers and patients, as well as the MMC WeChat account and official MMC WeChat public account, support real-time dynamic data upload for both in-hospital and remote patient monitoring. This integration facilitates comprehensive data processing across multiple platforms and provides a precise, multi-role, full-course management tool for the accurate follow-up of metabolic diseases.

#### MMC patient management and follow-up methods/procedures

##### MMC patient management

The consideration of various factors, such as personalized guidance on medication, dietary control, exercise, and psychological support, further emphasizes the importance of MMC healthcare collaboration.

**Figure 1:**
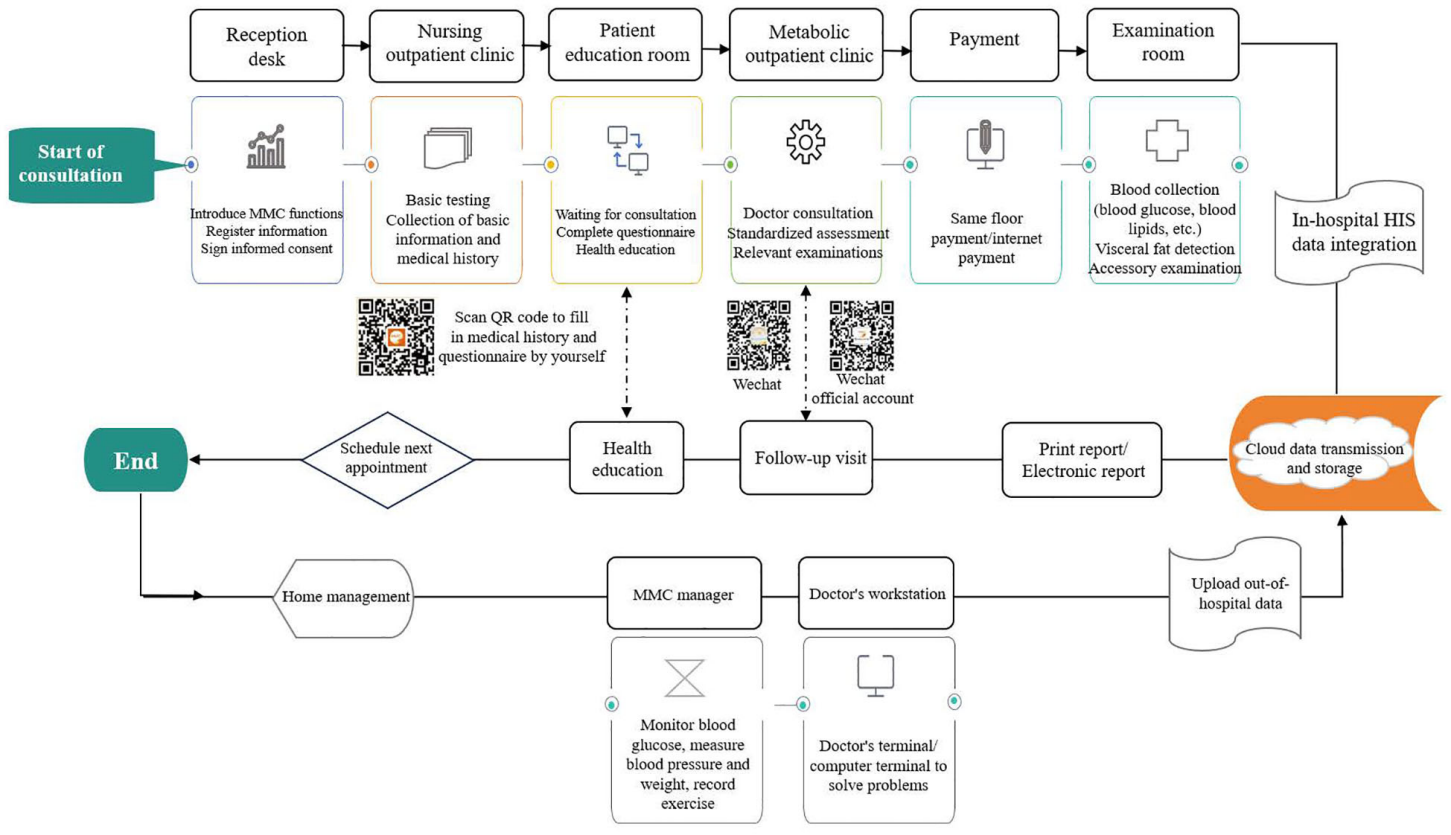
“One-stop” hospital management and internal and external management flow chart.

The MMC layout and workflow follow the SOP, enabling patients to complete all consultations in a single area. This approach eliminates the need to visit multiple examination rooms and establishes a centralized, one-stop service management system (**Figure 1**).

##### MMC follow-up management

According to the follow-up SOP, patients at different stages follow distinct diagnostic and follow-up procedures. Follow-up reminders are provided through two methods: 1) automatic reminder messages sent every three months via the follow-up system in the MMC data management platform; 2) telephone reminders through the MMC dedicated hotline based on the patient’s condition. During home care, follow-up management includes educational resources through the MMC WeChat service account, close connection via the app to monitor blood glucose warning values, providing patient guidance, and conducting telephone follow-ups.

**Figure 2:**
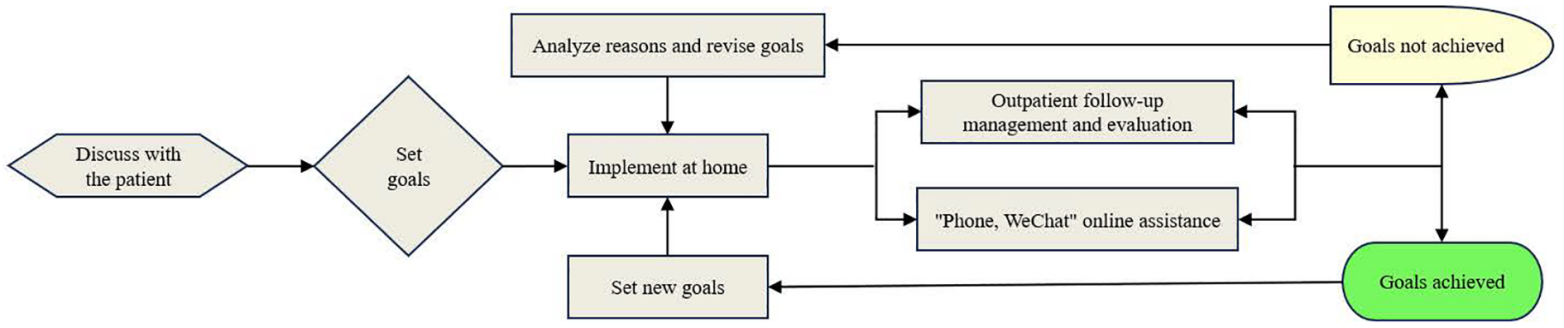
Flow chart of MMC with doctor-nurse collaboration.

By implementing a collaborative MMC process, the program ensures the efficient allocation of medical resources, facilitates effective teamwork among healthcare professionals, and provides personalized, continuous diagnostic and follow-up management. This approach enhances the prevention and treatment of metabolic diseases (**Figure 2**).

### Data collection and evaluation indicators

Upon entering the standardized MMC management, general data collection and measurements were performed on the first day, including age, gender, education level, disease duration, employment status, blood pressure, weight, body mass index (BMI), and waist circumference (WC). Laboratory indicators were measured on the second day of MMC entry and again one year after the intervention, including fasting plasma glucose (FPG), glycated hemoglobin (HbA1c), triglycerides (TG), total cholesterol (TC), high-density lipoprotein cholesterol (HDL-C), low-density lipoprotein cholesterol (LDL-C), and visceral fat. Additionally, a lifestyle survey was conducted on the first day of MMC entry and one year after the intervention to assess diet, exercise, smoking habits, and management satisfaction. The lifestyle questionnaire was based on the revised standardized Food Frequency Questionnaire (FFQ) (11, 12).

Responses were standardized and submitted on the same day through the MMC system. Patient satisfaction was measured using a simple questionnaire based on a Likert scale (0 to 3; 0 = not satisfied, 1 = average, 2 = satisfied). Patients rated their satisfaction with the consultation process before entering the MMC and again one year later to evaluate their experience.

Research staff informed patients about the study process, obtained signed informed consent, and assisted in filling out and submitting the relevant information. General data, medical history, laboratory results, and related disease information were collected, and satisfaction scores were recorded in the study form on-site. After approval from management at various levels, the relevant data for the study subjects were downloaded for processing and analysis.

### Statistical analysis

Data analysis was performed utilizing SPSS 26.0 statistical software, and GraphPad Prism 8 was used for plotting. Categorical data were expressed as frequencies and percentages, with comparisons before and after the intervention using the chi-square test. Normally distributed continuous data were expressed as means ± standard deviations ( $\bar{x}$ ± s), and paired t-tests were used for comparisons before and after management. Among them, the observation TG values is the paired T-test conducted after the natural logarithm is transformed into a normal distribution.

## Results

### General information of patients

The study included 452 diabetes patients. The distribution of gender, age, disease duration, education level, retirement status, and activity energy levels are shown in **Table 1**.

**Table 1:** General information of patients (n=452).

Project	Group	Frequency	Composition ratio (%)
Gender	female	164	36.28
	male	288	63.72
Age	18-35	58	12.83
	36-59	205	45.35
	60 and above	189	41.82
Disease duration (years)	0-5	180	39.82
	6-10	92	20.35
	11-20	130	28.76
	over 20	50	11.06
Education	Education below high school	149	32.96
	High school education or above	303	67.04
Retirement status	On the job	227	50.22
	retire	225	49.78
Activity/Energy Level	Mild physical strength	280	61.95
	Moderate physical strength	129	28.54
	Severe physical strength	43	9.51

### Comparison of weight and body fat-related indicators before and after MMC management

After one year of standardized management at the center, patients showed improved weight and BMI, with reductions in WC and visceral fat. The differences before and after the intervention were statistically significant (*P*<0.05), as shown in **Table 2**.

**Table 2:** Comparison of weight-related indicators before and after 1 year of collaborative MMC management (n=452, $\bar{x}$ ± s).

Item	Baseline	MMC Standardized Management 1 Year	P Value
Weight (kg)	76.19 ± 14.01	75.12 ± 13.80	<0.0001
BMI (kg/m²)	26.33 ± 3.81	25.97 ± 3.79	<0.0001
WC (cm)	94.62 ± 10.51	93.43 ± 10.39	<0.0001
Visceral Fat (cm²)	115.23 ± 49.41	105.66 ± 45.26	<0.0001

### Comparison of blood glucose and glycated hemoglobin before and after MMC management

**Figure 3:**
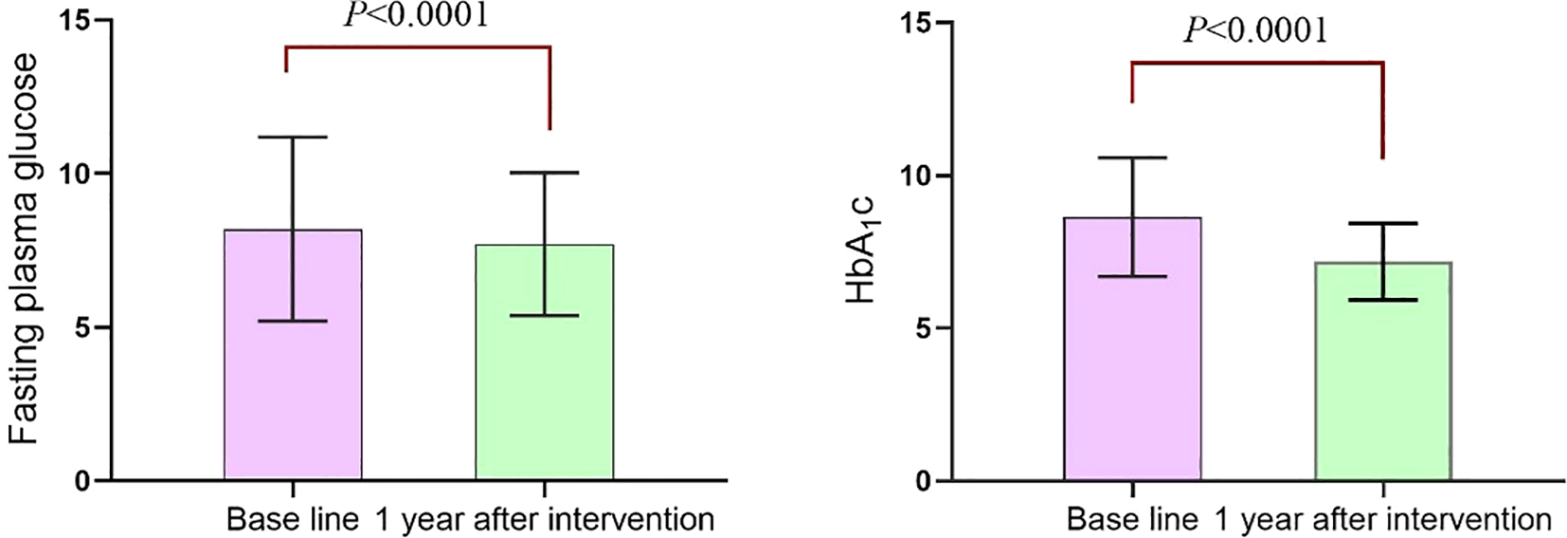
Comparison of fasting blood glucose and glycosylated hemoglobin.

After one year of standardized management at the center, patients exhibited a significant reduction in fasting blood glucose levels [(8.19 ± 2.99) mmol/L vs (7.71 ± 2.32) mmol/L] (*P*<0.0001). Similarly, glycated hemoglobin levels significantly decreased [(8.64 ± 1.95)% vs (7.18 ± 1.25)%] (*P*<0.0001), as shown in **Figure 3**.

### Comparison of blood pressure and blood lipid-related indicators before and after MMC management

**Figure 4:**
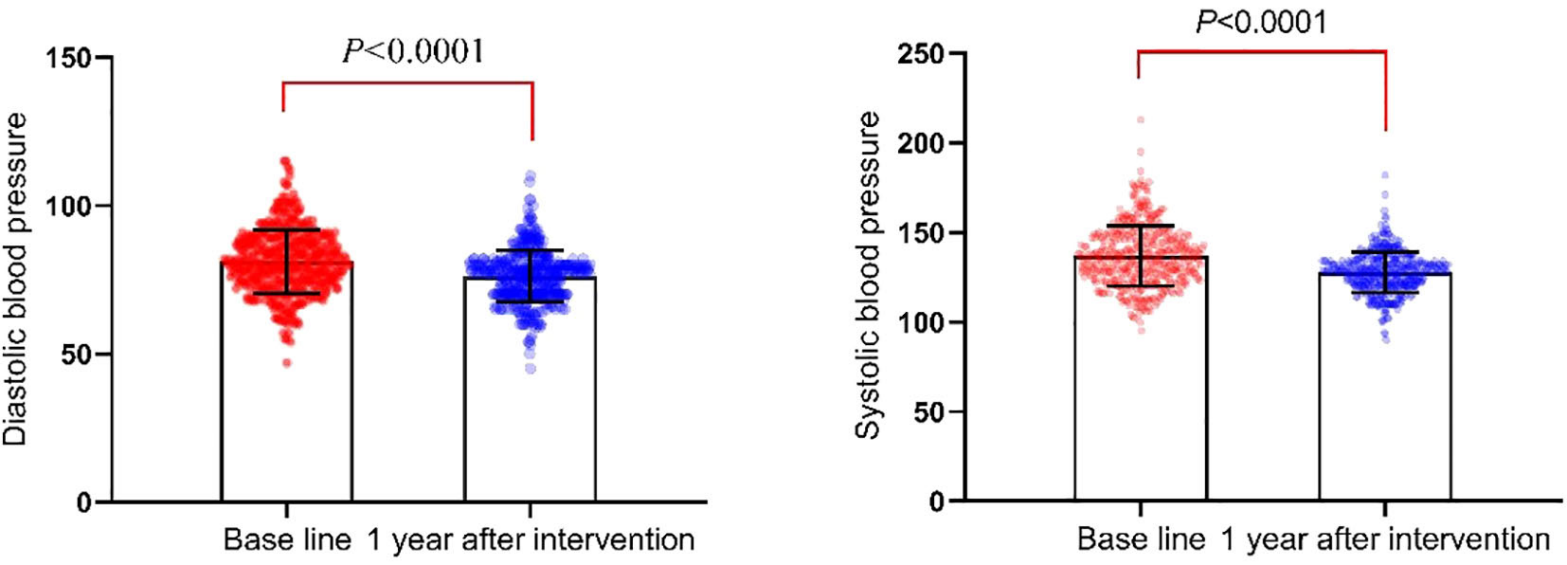
Comparison of systolic and diastolic blood pressure before and after MMC management.

After one year of standardized management at the center, patients showed significant reductions in blood pressure. Systolic blood pressure decreased from (136.92 ± 16.80) mmHg to (127.60 ± 12.63) mmHg (*P*<0.0001), and diastolic blood pressure decreased from (81.15 ± 10.62) mmHg to (76.05 ± 9.45) mmHg (*P*<0.0001), as illustrated in **Figure 4**.

**Figure 5:**
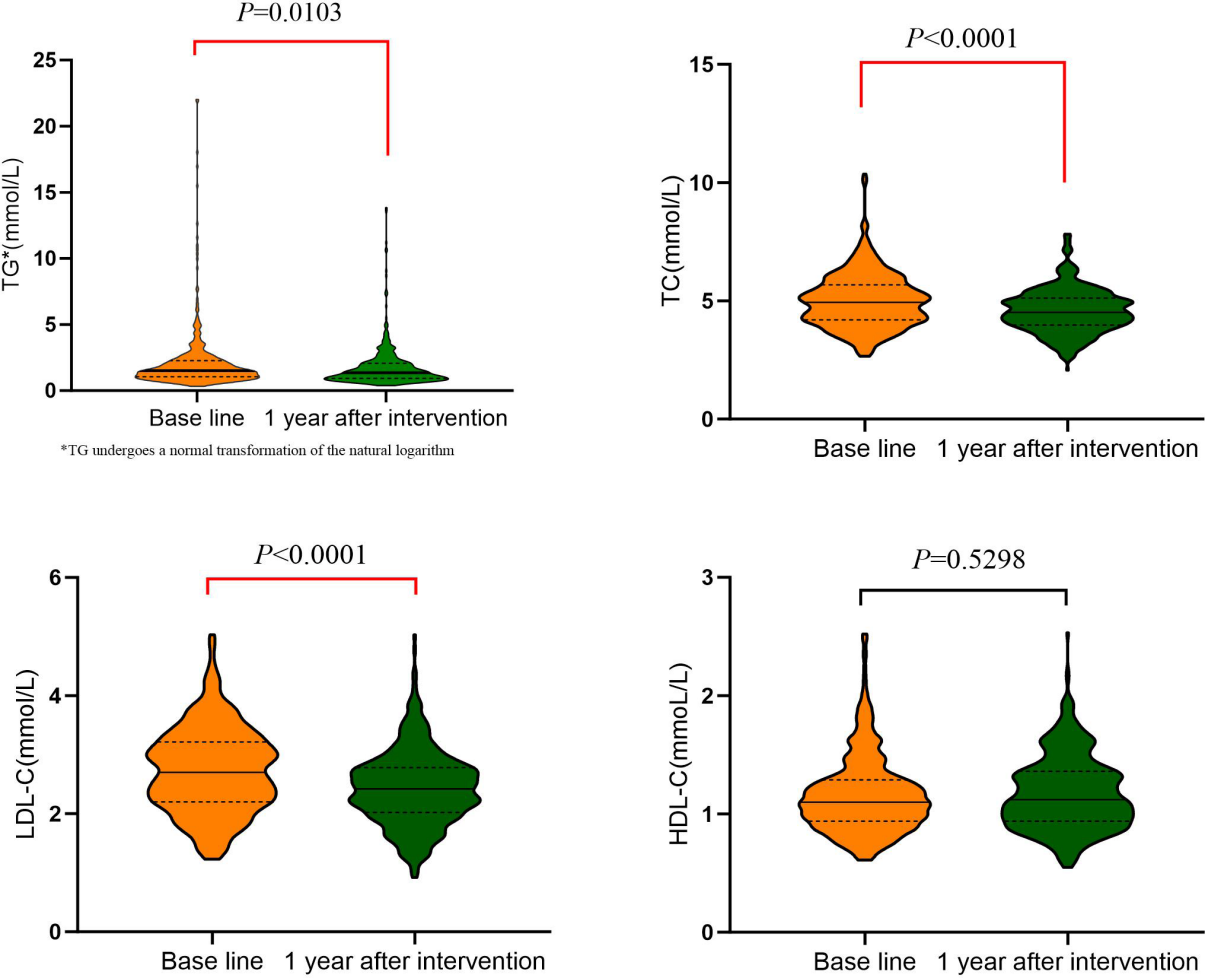
Comparison of blood lipid-related indicators before and after MMC management.

Blood lipid-related indicators also showed significant improvements. TG decreased from (2.12 ± 2.41) mmol/L to (1.77 ± 1.58) mmol/L (*P*=0.0103), TC decreased from (5.00 ± 1.13) mmol/L to (4.57 ± 0.92) mmol/L (*P*<0.0001), and low-density lipoprotein cholesterol (LDL-C) decreased from (2.73 ± 0.71) mmol/L to (2.43 ± 0.63) mmol/L (*P*<0.0001). However, there was no significant change in HDL-C, which remained at (1.16 ± 0.325) mmol/L before and (1.17 ± 0.308) mmol/L after the intervention (*P*=0.5298), as displayed in **Figure 5**.

### Comparison of diet, exercise behavior, and satisfaction before and after MMC management

After one year of standardized health guidance from MMC, patients showed significant improvements in their daily intake of vegetables and fruits, salt consumption, sugary drink intake, and weekly fish consumption (*P*<0.05). However, the difference in salt intake was not statistically significant (*P*>0.05). Additionally, weekly exercise and smoking cessation behaviors showed statistically significant improvements (*P*<0.05). Compared to regular outpatient visits, satisfaction with medical care after one year of MMC management also showed a statistically significant increase (*P*<0.05), as shown in **Table 3**.

**Table 3:** Comparison of lifestyle and satisfaction before and after 1 year of collaborative MMC management (n=452, n (%).

Item	Baseline	MMC standardized management 1 year	P Value
Daily vegetable intake ≥ 400g	216 (47.79%)	261 (57.74%)	0.003
Daily fruit intake ≥ 200g	175 (38.72%)	312 (69.03%)	0.013
Daily salt intake ≤ 5g	113 (25.00%)	120 (26.55%)	0.648
Weekly fish intake ≥ 2 times (each >100g)	255 (56.42%)	300 (66.37%)	0.003
No or occasional sugary drink consumption	371 (82.08%)	439 (97.12%)	0.000
Exercise ≥ 2 times per week (each ≥ 10 mins)	281 (62.17%)	386 (85.40%)	0.000
Non-smokers or quit smoking	297 (65.71%)	330 (73.01%)	0.021
Patient satisfaction	2.53 ± 0.50	2.86 ± 0.35	0.000

## Discussion

Weight management is a key aspect of comprehensive type 2 diabetes care. Clinical practice should emphasize a gradual, sustained approach, focusing on controlling weight, reducing BMI, decreasing visceral fat, and tracking changes in waist circumference. Waist circumference is a simple and practical indicator for assessing visceral fat and cardiovascular metabolic risk (3, 13, 14). In this study, patients exhibited significant reductions in weight, BMI, and waist circumference after one year of collaborative MMC management, demonstrating effective weight management. The success can be attributed to the collaborative efforts between healthcare providers and patients in setting realistic weight control goals. Physicians tailored glucose-lowering medications to support weight reduction based on individual patient needs. Nurses analyzed factors contributing to overweight or obesity, educating patients on balanced diets with caloric restrictions and providing personalized exercise guidance. Monitoring weight to prevent fluctuations and informing patients about the importance of weight management in controlling blood glucose, especially for obese patients, was emphasized. Effective weight control aids in blood glucose management, with a 3% to 5% reduction in weight yielding significant clinical benefits (15, 16). The collaborative approach, involving standardized use of weight-reducing medications, balanced diets, and exercise, is vital for achieving and maintaining long-term weight reduction.

Research has confirmed that maintaining optimal blood glucose levels is essential for preventing and managing diabetic complications (17). HbA1c remains the “gold standard” for assessing whether blood glucose control in diabetes patients is sufficient (18, 19). In this study, after one year of standardized collaborative MMC management, patients showed significant improvements in FPG and HbA1c levels, decreasing from (8.19 ± 2.99) mmol/L to (7.71 ± 2.32) mmol/L and from (8.64 ± 1.95)% to (7.18 ± 1.25)%, respectively. The improvements can be attributed to the standardized, one-stop management approach implemented by the MMC through healthcare collaboration. During home self-management, the MMC team conducted regular follow-ups, with nurses routinely contacting patients to monitor their conditions and sending appointment reminders through text messages and follow-up calls. Physicians addressed abnormal blood glucose levels via the “Doctor’s Workstation” platform, while patients uploaded their blood glucose, blood pressure, and exercise data through the “MMC Manager” app. Patients could also reach the center via WeChat or phone calls to address any issues encountered at home. The National Standardized Metabolic Disease Management Center’s scientific and standardized processes, implemented and monitored through healthcare collaboration, allow for the timely identification of issues and necessary improvements and adjustments. This collaborative approach enhances effective coordination among healthcare providers and comprehensive patient management, significantly boosting patient compliance and confidence and improving blood glucose control outcomes.

Experts emphasize the importance of integrated management for hypertension, diabetes, and dyslipidemia to ensure standardized control of these conditions (20). Diabetes is an independent risk factor for cardiovascular disease and often coexists with other major cardiovascular risk factors such as hypertension and dyslipidemia (21). Research has shown that effective control of blood lipids and blood pressure can help manage the progression of diabetic retinopathy (3). In this study, the MMC healthcare team followed SOPs, considering patient age, disease duration, complications, and Atherosclerotic Cardiovascular Disease (ASCVD) risk stratification to develop appropriate antihypertensive and lipid-lowering medication plans. Nurses guided patients in monitoring their blood pressure, provided information on medication usage, and set ideal target values, improving patient awareness and adherence. Studies have demonstrated that modest weight loss can help control blood glucose and improve blood pressure, blood lipid levels, and cardiovascular outcomes (22–24). After one year of intervention, patients in this study showed a decrease in weight and improvements in blood pressure and non-HDL-C levels, with no cardiovascular events occurring during the period. These findings are consistent with Yang Jie et al. (25). After one year of intervention, patients in this study showed a decrease in weight, along with improvements in blood pressure and non-HDL-C levels, with no cardiovascular events occurring during the period. These findings align with those of Yang Jie et al. (25). HDL-C levels remained stable before and after the intervention. Although HDL-C is recognized as an important factor in cardiovascular disease, especially ASCVD, LDL-C and non-HDL-C remain the primary targets for lipid-lowering in ASCVD risk management. Some studies suggest that increasing HDL-C levels through medication does not positively prevent cardiovascular events or reduce risks (26, 27). Critical factors contributing to decreased HDL-C levels include poor dietary habits, smoking, alcohol consumption, and sedentary lifestyles. Non-pharmacological interventions, particularly lifestyle changes, are the key determinants in preventing ASCVD (28, 29). For diabetes patients, lifestyle interventions form the foundation for lipid management, helping lower blood lipid levels while benefiting blood pressure, blood glucose, and overall cardiovascular health. The ideal control of these indicators is consistent with the latest guidelines from the American Diabetes Association (ADA) on cardiovascular disease and risk management (30). Future long-term follow-up studies will further evaluate the potential impact of this management model on patients’ long-term metabolic health and cardiovascular disease risk reduction.

Improving self-management skills in diabetes patients is crucial for controlling blood glucose and preventing complications. Enhancing self-management involves improving lifestyle and behavioral habits. Du et al. (25, 31) demonstrated that the MMC management model effectively improves patients’ self-management abilities. In this study, the collaborative MMC management intervention significantly impacted patients’ dietary behaviors, exercise habits, smoking, and overall satisfaction. After one year of intervention, the intake of fresh fruits and vegetables increased significantly compared to the baseline. The percentage of patients who did not drink or only occasionally drank sugary beverages rose from 80.08% to 97.12%. Weekly fish intake of at least two servings (more than 100g per serving) improved significantly, meeting dietary guidelines recommending at least two servings of fish per week (300-500g). This increase in fish consumption, rich in low fat and beneficial fatty acids, helps protect the cardiovascular system, prevent complications, and improve nutritional balance and immunity in diabetes patients. The percentage of patients exercising at least twice a week (for at least 10 minutes per session) rose from 62.17% to 85.40%. However, no significant improvement was observed in daily salt intake of 5g or less from baseline to one year later. The study participants resided in northeastern China, where local dietary habits are characterized by strong flavors and high salt intake. Salt consumption is influenced by factors such as the number of people in the household and whether meals are prepared at home or in restaurants. Given these factors, it is difficult to change patients’ dietary habits in a short time. Future research should focus on developing more targeted intervention strategies for these indicators and emphasize the need for strict management of salt intake. In addition to diet and exercise, smoking is an independent risk factor for elevated glycated hemoglobin levels (32) and increases the risk of diabetes complications, especially macrovascular diseases. Studies have shown that the risks of coronary heart disease, stroke, and myocardial infarction increase by 54%, 44%, and 52%, respectively, along with a 48% increase in all-cause mortality risk (33). In this study, the percentage of patients who either never smoked or quit smoking increased from 65.71% at baseline to 73.01% after one year of intervention, showing significant improvement. Smoking cessation can help control blood glucose, improve blood lipid levels, and significantly reduce the incidence of cardiovascular diseases (34, 35). Several studies support smoking cessation in reducing the incidence of coronary heart disease and the progression of diabetic nephropathy. Smoking cessation for more than six years can reduce the risk of cardiovascular disease and mortality in diabetes patients (36–38). Additionally, patient satisfaction with healthcare visits improved after one year of intervention in this study.

The MMC management model is based on actively learning from international diabetes management experiences while considering the unique characteristics of diabetes in contemporary China. The model follows the concept of “one center, one-stop service, one standard” and involves multidisciplinary collaboration, including medical professionals and healthcare providers, to create an advanced and successful diabetes management model with Chinese characteristics (39, 40). Representative international models, such as the National Health Service (NHS) management model (41) and patient-centered primary care models, emphasize patient engagement and self-management. Multidisciplinary teams provide comprehensive support to improve patients’ quality of life and achieve significant improvements in blood glucose control. Preventive management models focus on early prevention and controlling risk factors to reduce diabetes incidence. However, these models tend to be less effective for managing patients already diagnosed with diabetes. In contrast, well-developed community health services have applied chronic care models (42), empowering supporters to jointly manage care (43), and shared medical appointment models (44), which have proven effective in managing blood glucose, blood pressure, promoting health behavior changes, and identifying care barriers through telemedicine. Compared to international models, the MMC management model integrates innovations such as “Internet+” and multidisciplinary collaboration, making it better suited to China’s healthcare system. Although the MMC framework differs from the six core components of the diabetes care plans commonly described in the American Diabetes Association’s Chronic Care Model (CCM), it shares similar goals. This study validates the effectiveness of the hospital-based approach, while DSMES (Diabetes Self-Management Education and Support) remains a key intervention and the foundation for successfully implementing CCM. A collaborative, professional team approach is optimal for managing chronic diseases like diabetes and promoting self-management. Future improvements, supported by high-performance computing (HPC) and artificial intelligence (AI), will enhance the model’s effectiveness. Combined with tiered healthcare and more precise execution, these innovations will enable deeper comparisons of the strengths and weaknesses of different models, providing stronger evidence for optimizing diabetes management in China (45, 46).

As an exploratory study, this research aims to assess whether patients benefit from the “one-stop diagnosis and management model” before and after its implementation. Convenience sampling with a quasi-experimental, within-subject design was used, which introduces selection bias. The non-random allocation method is prone to selection bias, potentially weakening the strength of the conclusions. To reduce bias and improve the reliability of the findings, the sample size was increased to enhance representativeness. Future studies will use a randomized controlled design to minimize the impact of selection bias. This study represents an initial exploratory investigation, with subsequent research planned to evaluate the effects of this management model on metabolic indicators and long-term cardiovascular risk, providing stronger evidence for causal relationships. Lifestyle data in this study were self-reported, which may introduce recall bias or social desirability bias. Future research could incorporate objective measurement tools, such as fitness trackers, dietary logs, and wearable devices for step counting, to enhance the reliability of the data.

## Conclusions

The collaborative healthcare model established at the National Standardized Metabolic Disease Management Center is built on scientific, standardized, practical, and assessable principles. Collaborative healthcare, a central tenet of modern medical practice, emphasizes the close coordination among doctors, nurses, and other healthcare professionals, underscoring its significance. By implementing this collaborative approach, the National Standardized Metabolic Disease Management Center achieves rational allocation of medical resources, effective teamwork, and comprehensive patient management. The model has proven effective in managing weight-related indicators in diabetes patients, significantly lowering blood glucose, glycated hemoglobin levels, blood pressure, and blood lipids. It has also improved patients’ behavioral habits, contributing to better prevention and control of metabolic diseases and treatment outcomes while increasing patient satisfaction.

## Data Availability

The datasets presented in this study can be found in online repositories. The names of the repository/repositories and accession number(s) can be found in the article/supplementary material.
